# Radiation Recall Reaction Induced by Adjuvant Trastuzumab (Herceptin)

**DOI:** 10.1155/2009/307894

**Published:** 2009-09-08

**Authors:** Caroline Chung, David Stuart, Mira Keyes

**Affiliations:** ^1^Department of Radiation Oncology, BC Cancer Agency, University of British Columbia, Vancouver, Canada V5Z 4E6; ^2^Department of Medical Oncology, Burnaby General Hospital, Burnaby, Canada V5G 2X6

## Abstract

Although concerns of radiation sensitization have been raised with concurrent trastuzumab (Herceptin) administration, there has been no published case of radiation recall reaction associated with trastuzumab. This case describes a clinical presentation consistent with a radiation recall reaction following administration of adjuvant trastuzumab after neoadjuvant FEC-D chemotherapy and locoregional radiotherapy for HER2-positive, locally advanced breast cancer in a premenopausal woman. Although the mechanism and etiology of radiation recall dermatitis remain unclear, this case raises further hypotheses regarding a possible drug dose-dependence and possible predisposing risk factor for the development of radiation recall reactions.

## 1. Presentation of Case

A 41-year-old premenopausal woman presented with slight discomfort in the left breast and a large palpable mass tethered to the underlying muscle in the left breast along with a small palpable node in the left axilla. Mammographically, a 5 × 5 cm mass was noted in the upper outer quadrant of the left breast. Her previous mammogram a year ago was negative. The fine needle aspirate of the breast mass was positive for grade 3 invasive ductal carcinoma, which was ER negative, PR negative, and Her2/neu amplified on FISH analysis. Her metastatic work up, including a liver ultrasound and bone scan, was negative. She was therefore clinically staged as T3N1MX [1].

Her previous medical history included eczema, allergic rhinitis, and contact dermatitis to numerous allergens, including latex. She was otherwise well. Her only previous surgeries were tubal ligation and tonsillectomy. Her breast cancer specific history included menarche at age 11 with long-term oral contraceptive use from age 12 to 26 for menstrual regulation. She was nulliparous. Her menses stopped shortly after starting chemotherapy but restarted in April 2006. Her family history was unavailable as she was adopted and did not have information about her biological parents.

Given her presentation with locally advanced breast cancer, she received neoadjuvant FEC-D chemotherapy, consisting of 3 cycles of 5-fluorouracil, epirubicin, and cyclophosphamide then 3 cycles of docetaxel. She had a clinical complete response after her first cycle of FEC. She tolerated all 3 cycles of FEC reasonably well. However, after her first cycle of docetaxel, she developed a severe peripheral neuropathy with significant weakness and neuropathic pain in her upper and lower extremities such that she was unable to walk for about a week. This was more severe on the left side than the right. As a result, the subsequent 2 cycles of docetaxel were reduced in dose by 50%. 

Following completion of chemotherapy, she received locoregional radiotherapy directed to the breast, internal mammary chain (IMC) lymph nodes, and axilla. She was treated with CT planned 4-field radiotherapy using wide tangents to encompass the left breast and IMC lymph nodes along with anterior and posterior axillary fields to encompass the axillary nodal regions (axillary levels I, II, and III nodal regions and the supraclavicular and infraclavicular nodal regions). The whole left breast received 4250 cGy in 16 fractions delivered by photons followed by a boost of radiation (1000 cGy in 5 fractions) to the initial tumour location with a margin, delivered by electrons. The locoregional lymph nodes received 3750 cGy in 16 fractions. 

During the course of radiotherapy, she developed brisk skin erythema throughout the breast with moist desquamation in the inframammary breast fold, requiring both topical betamethasone and flamazine cream. The skin healed gradually over the month following completion of radiotherapy. She was offered left breast upper outer quadrantectomy and axillary lymph node dissection following her recovery from radiotherapy, which she declined.

Several weeks after completing radiotherapy but prior to starting trastuzumab, she developed a severe constant squeezing left-sided chest pain, which lasted about an hour. This developed while at rest in bed, and it was so severe that she was bed-ridden for the entire episode, but the pain subsided spontaneously without any intervention. She went for an ECG, bloodwork, and chest x-ray within the next few days, which were all normal. However, she continued to notice a vague heavy sensation in her chest, which limited her activity, particularly walking up stairs. There was no associated shortness of breath or cough.

She agreed to proceed with trastuzumab therapy starting 4 weeks after completing radiotherapy for a total of 17 cycles. She completed eleven doses of trastuzumab 513.28 mg intravenously in the right (contralateral) arm with which she was experiencing subtle, intermittent left chest wall discomfort, not interfering with her daily activities. As she was concerned about the potential long-term toxicities of repeat Trastuzumab given in one arm, she requested the 12th cycle be given via the left (ipsilateral) arm. 

Three days after receiving trastuzumab in her ipsilateral arm, she awoke with a painful, swollen, and erythematous left breast, axilla and medial left upper arm, which resembled her previous radiation dermatitis reaction. The erythematous region of the skin was very well demarcated, resembling the area of irradiated skin with sharp linear borders following the borders of her previous radiation fields. The brisk erythema largely resolved spontaneously within 2 days, but the edema and dull, throbbing ache in the breast and chestwall persisted for about 2 weeks. As this occurred during the weekend, she did not seek medical attention immediately and was seen by the medical oncologist a few days after the skin reaction had subsided.

At the time of assessment by Radiation Oncology, about 2 weeks after the initial recall reaction, nearly a year since completing her radiotherapy, she had 2 areas of persistent tenderness: one in the left inframammary fold and the other in a more inferior lateral rib. These 2 areas had initially become tender during her radiotherapy treatment and then had settled. Subsequently, she had intermittent, exacerbations of discomfort in these areas during her trastuzumab treatment, but the pain was not as severe as during the “radiation recall” reaction. At the time of assessment, these areas were only mildly tender. A bone scan investigating the rib pain did not show any uptake representing malignancy or trauma. 

Despite the recall reaction, she continued with the planned doses of trastuzumab, 513.28 mg intravenously via the right (contralateral) arm every three weeks, until completion of all 17 cycles. She did not receive any premedication with steroids or antihistamines. She completed the remainder of the cycles without any further episodes of radiation recall dermatitis, but she continued to experience vague, intermittent left chestwall discomfort, as she had noted prior to the recall reaction.

## 2. Discussion

Over-expression of human epidermal growth factor receptor type 2 (HER2) is seen in 20%–30% of invasive breast carcinomas. Trastuzumab is a recombinant monoclonal antibody with the capability of binding to two antigen-specific sites on the HER2 receptor, which in turn is thought to prevent activation of the intracellular tyrosine kinase domain. HER2 downstream signaling is believed to promote cellular proliferation and inhibit cell death [2]. However, mechanisms that may result in trastuzumab-related toxicity are not fully understood.

There have been several large randomized studies demonstrating improvement in overall survival and disease control with adjuvant trastuzumab after primary breast cancer therapy (surgery, adjuvant or neoadjuvant chemotherapy, with or without radiotherapy). Based on these findings, adjuvant trastuzumab therapy has been incorporated into the standard management of HER2 positive breast cancer in North America [3–5]. From these studies, the primary adverse effect of trastuzumab was cardiac toxicity with impairment of left ventricular ejection fraction. Other commonly reported toxicities include flu-like symptoms and cough, abdominal discomfort and diarrhea, and skin rash [6]. Belkacemi et al. recently reported concurrent trastuzumab and radiotherapy resulted in 51% grade ≥ 2 acute radiation dermatitis in a French multicentred study, which is a higher incidence than seen with adjuvant radiotherapy alone [7]. However, there have been no previous cases of trastuzumab-associated radiation recall reactions. Based on previous reviews on radiation recall dermatitis, this case presentation is consistent with a radiation recall dermatitis associated with trastuzumab.

Radiation recall dermatitis is a rare complication associated with exposure to a recall-triggering drug after radiation treatment, which is poorly understood. In order for the acute reaction to be classified as a radiation-recall reaction, the recall-triggering drug should be given at least 7 days after radiotherapy. Otherwise, the reaction may be due to a radiosensitization effect, as the drug interferes with the cellular repair mechanisms at work after radiation exposure. In a recent review of the published case reports on radiation recall dermatitis, the median interval between radiation and the recall-triggering drug was found to be 39.5 days. In the same review, the median speed of onset of the recall reaction was 3 days for intravenous drugs, and the time taken for resolution of the reaction ranged from hours to 14 days [8]. The primary effective treatment for radiation recall dermatitis was discontinuation of the drug. All other attempted treatments, including topical/oral steroids or antihistamines, have not demonstrated any effect on the resolution of the reaction. When patients were rechallenged with the recall-triggering drug, the recall dermatitis did not consistently recur. Based on the clinical presentation of radiation recall dermatitis with a rapid onset, rare incidence, and unpredictable effect of drug rechallenge, the most plausible possible mechanism is an idiosyncratic drug hypersensitivity reaction, rather than any cytotoxic effect of the drug that triggered the reaction [9]. In further support of this mechanism, there have been a wide range of drugs associated with radiation recall dermatitis from the most common being adriamycin and paclitaxel to the very rarely associated drugs such as TB medications and Tamoxifen [10–14]. The new targeted therapies such as cetuximab, a monoclonal antibody to epidermal growth factor receptor, have only been associated with acneiform skin rash until recent reports of radiation recall phenomenon and increased radiation dermatitis [15, 16]. 

This case presents with a very typical presentation of radiation recall dermatitis following a dose of trastuzumab, which has not previously been associated with radiation recall. The clinical distribution of the skin reaction following the geometrical borders of the radiation field, the time to onset of 3 days, and the time to spontaneous resolution of 14 days are all consistent with previous reports of radiation recall dermatitis. There are several additional interesting facets to this case, which pose further questions and possible mechanisms for radiation recall reactions. This lady's documented history of skin hypersensitivity to numerous allergens including latex raises the question of whether this may predispose a person to developing radiation recall dermatitis. Secondly, as the recall dermatitis only developed after she received her trastuzumab infusion in the ipsilateral arm to which she received radiotherapy, there may be a dose-dependent response and a possible threshold dose exposure that triggers a radiation recall reaction. Similar to previous cases, this lady was able to complete the remainder of her planned trastuzumab treatment without another recall reaction, although a rechallenge in the ipsilateral arm was not attempted. 

Clearly, the mechanism and etiology of radiation recall dermatitis remain unclear. The rarity of these reactions and the heterogeneous presentations of cases add to the challenges in further characterizing and understanding this phenomenon.

## 3. Consent

Informed consent was obtained from the patient for publication of this case report.
